# A novel nonsense mutation in *TNNT2* in a Chinese pedigree with hypertrophic cardiomyopathy: A case report

**DOI:** 10.1097/MD.0000000000021843

**Published:** 2020-08-21

**Authors:** Guangyuan Gao, Guohui Liu, Weiwei Chen, Yaliang Tong, Cuiying Mao, Jinsha Liu, Xing Zhang, Max M. He, Ping Yang

**Affiliations:** a Department of Cardiology, China-Japan Union Hospital of Jilin University; b Jilin Provincial Key Laboratory for Genetic Diagnosis of Cardiovascular Disease, Changchun; c Simcere Diagnostics Co., Ltd., Nanjing, China.

**Keywords:** hypertrophic cardiomyopathy, nonsense mutation, *TNNT2*, whole exome sequencing

## Abstract

**Rationale:**

Hypertrophic cardiomyopathy (HCM) is an inherited myocardial disease and a common cause of sudden cardiac death, heart failure, atrial fibrillation and stroke. In families affected by HCM, genotyping is useful for identifying susceptible relatives. In the present study, we investigated the disease-causing mutations in a three-generation Chinese family with HCM using whole exome sequencing (WES).

**Patient concerns:**

The proband, a 50-year-old man, was diagnosed with HCM at the age of 41 years. He presented with an asymmetric hypertrophic interventricular septum and a maximum interventricular septum thickness of 18.04 mm. His third elder sister, niece and daughter were also clinically affected by HCM.

**Diagnosis:**

Autosomal dominant HCM.

**Interventions:**

Seven family members, including 4 affected members, accepted WES. The genetic variants were subsequently called using Genome Analysis Toolkit and annotated using the InterVar program. Following frequency filtration by the Genome Aggregation Database, the variants were evaluated using an in-house bioinformatics analysis pipeline.

**Outcomes:**

HCM was transmitted as an autosomal dominant trait in the family. An extremely rare stop gained mutation, rs796925245 (g.1:201359630G>A, c.835C>T, p.Gln279Ter) in the troponin T2 (*TNNT2*) gene was identified as the disease-causing mutation. The stop gained mutation was predicted to result in a truncated troponin T protein in cardiac sarcomere. An adolescent family member who had normal echocardiographic measurements was found to carry the same disease-causing mutation.

**Lessons:**

A novel nonsense *TNNT2* mutation was identified as the HCM-causing mutation in this Chinese pedigree. Since HCM shows a low penetrance by clinical criteria in adolescents, the adolescent mutation carrier, who is still clinically unaffected, should be offered routine follow-ups and sport activity recommendations to prevent adverse events including sudden cardiac death in the future.

## Abstract 2

Supplemental Digital Content is available in the text

## 1 Introduction

Hypertrophic cardiomyopathy (HCM) is an inherited myocardial disease characterized by myocardial hypertrophy in the absence of excess external load.^[1,2]^ It is a common cause of heart failure, atrial fibrillation, stroke and sudden cardiac death in young people.^[3,4]^ The prevalence of HCM is 0.2% in the general population and 0.08% in the Chinese adult population.^[3,5]^

HCM is genetically heterogeneous and caused by more than 1400 mutations in cardiac sarcomere or Z-disc protein genes, most of which are unique to individual families.^[3]^ In clinical practice, the diagnosis of HCM is based on the detection of increased left ventricular wall thickness by echocardiography or cardiovascular magnetic resonance imaging.^[4]^ However, many functional changes in HCM, including altered calcium cycling and sensitivity, disturbed stress sensing and impaired cardiac energy homeostasis are clinically undetectable.^[6]^ Additionally, since HCM shows an age-related penetrance, many mutation carriers do not have typical symptoms or left ventricular hypertrophy in imaging findings early in life.^[1]^ Therefore, genetic testing is useful for susceptible relative screening in families affected by HCM.^[1,7]^ Furthermore, genetic testing represents a significant advancement in the understanding of HCM pathogenesis.^[8]^

Recently, whole exome sequencing (WES) has been increasingly used to detect the genetic basis of diseases by sequencing the protein-coding exome.^[9]^ It is an accurate and cost-effective method for genetic screening of very rare Mendelian disorders.^[9]^ Additionally, in comparison with conventional sequencing methods, WES does not need to make a priori assumptions associated with the causes of the disease.^[1]^

In the present study, WES was performed to determine the precise mutations that are associated with HCM in a three-generation Chinese family. The proband in this family is a middle-aged man. He was diagnosed with HCM 9 years ago and first admitted to our hospital presenting with chest distress and chest pain during exercise. His sister, niece and daughter were diagnosed with HCM at the age of 46, 22, and 18 years, respectively. It appears that HCM was transmitted as an autosomal dominant trait in this family. Genetic testing may help identify the genetic cause of HCM, provide accurate genetic counseling for unaffected members in this family, and expand the spectrum of HCM-causing mutations for further pathogenic studies.

## 2 Methods

### 2.1 Patients and subjects

The present study was approved by the Ethics Review Board of China-Japan Union Hospital of Jilin University (Changchun, China). All procedures, including blood storage and data collection were performed in accordance with the Declaration of Helsinki. Written informed consent was obtained from the patients for publication of this case report and accompanying images before WES. The medical records and diagnosis of HCM were confirmed by two independent cardiologists. The function and structure of the hearts was assessed by transthoracic echocardiography and 12-lead electrocardiograms. HCM is defined by a maximum wall thickness of ≥15 mm in one or more left ventricular myocardial segments in echocardiography.^[1]^ Left ventricular outflow tract obstruction is defined as a peak instantaneous Doppler left ventricular outflow tract pressure gradient of ≥30 mmHg.^[1]^ Venous blood was drawn and collected in ethylenediaminetetraacetic acid-containing tubes for DNA extraction.

### 2.2 WES and bioinformatics analysis

Blood was centrifuged at 2000 × g for 10 minutes and the white blood cell layer was collected for DNA extraction. Genomic DNA was extracted using an automatic nucleic acid extractor (Thermo Fisher Scientific, Inc., Waltham, MA). Following the DNA quality inspection, 1 μg genomic DNA per blood sample was used to prepare the library for exons. Exons were captured using Sure Select Human All Exon V5 kit (Agilent, Santa Clara, USA) and sequenced by Illumina HiSeq 4000 sequencer (Illumina, Saint Diego, USA). FastQC software (version 0.11.8) was used to check the quality of the raw sequence. The clean data was then mapped to the human genome (NCBI37/hg19) using BWA software (http://sourceforge.net/projects/bio-bwa/). Single nucleotide polymorphisms (SNPs) and small insertions and deletions (INDELs) were called in Human Genome Variation Society (HGVS) using GATK software (version 4.1.0.0). InterVar program (http://wintervar.wglab.org/) was used to annotate all the variants.^[10]^ The minor allele frequency (MAF) for each variant was evaluated by the Genome Aggregation Database in the East-Asian population (http://gnomad.broadinstitute.org/). The effects of the variants on protein coding were evaluated using 12 programs, including SIFT,^[11]^ PolyPhen-2_HDIV,^[12]^ Polyphen2_HVAR,^[12]^ LRT,^[13]^ MutationTaster,^[14]^ MutationAssessor,^[15]^ FATHMM,^[16]^ PROVEAN,^[17,18]^ MetaSVM,^[19]^ MetaLR,^[19]^ M-CAP,^[20]^ and fathmm-MKL.^[21]^

## 3 Results

### 3.1 Clinical characteristics

**Figure 1:**
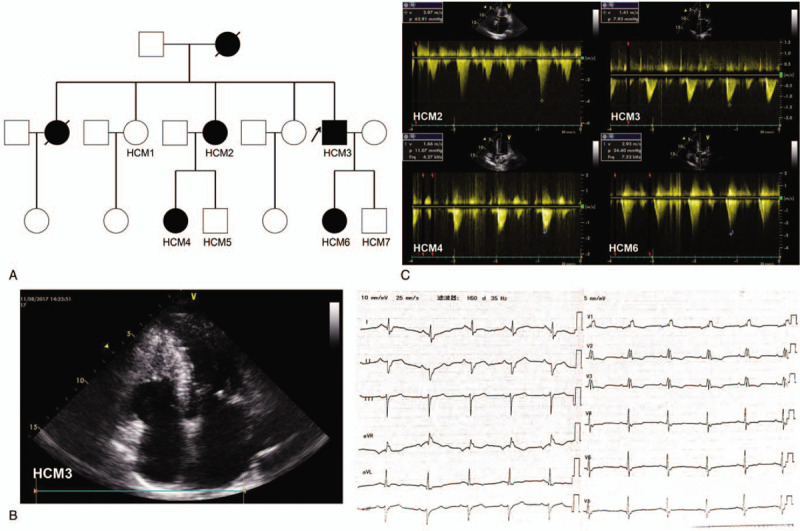
A. Pedigree of the family with HCM. HCM = hypertrophic cardiomyopathy; White square, normal male; white circle, normal female; black square, male patient with HCM; black circle, female patient with HCM; arrow, the proband; slash, deceased. B. B-mode echocardiogram (left) and 12-lead electrocardiogram (right) of HCM3. C. Doppler echocardiograms of HCM2, HCM3, HCM4 and HCM6. Left ventricular outflow tract pressure gradient of HCM2 and HCM6 was significantly increased. v = blood flow velocity through the left ventricular outflow tract; p = left ventricular outflow tract pressure gradient; Frq = returning sound wave frequency through the left ventricular outflow tract.

This three-generation Chinese family with HCM was from the north of China (Fig. 1A). The clinical characteristics of 4 HCM patients (HCM2, HCM3, HCM4, and HCM6) and 3 unaffected family members (HCM1, HCM5, and HCM7) are presented in Table 1.

**Table 1:**
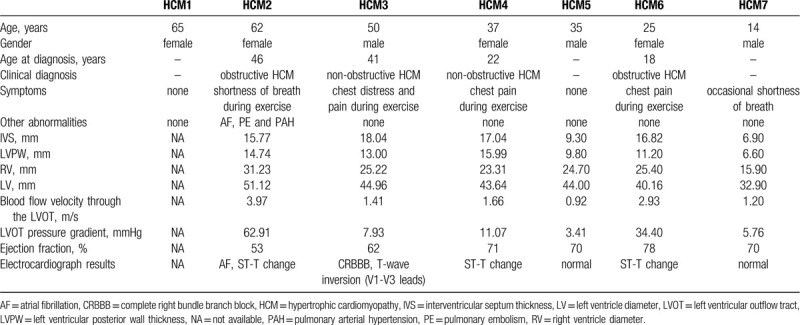
The Clinical Characteristics of the Hypertrophic Cardiomyopathy Pedigree.

The proband (HCM3 in Fig. 1A), a 50-year-old man, was diagnosed with HCM at the age of 41 years. He presented with an asymmetric hypertrophic interventricular septum and a maximum interventricular septum thickness of 18.04 mm (Fig. 1B and Table 1). He had been suffering from chest distress and chest pain during exercise for 9 years, which had worsened in the past 3 years. The electrocardiogram of HCM3 showed sinus rhythm, complete right bundle branch block and T-wave inversion in the anterior wall leads (Fig. 1B).

The proband's third elder sister (HCM2 in Fig. 1A) was 62 years old and diagnosed with HCM at the age of 46 years. She showed an increase in interventricular septum thickness (15.77 mm) and left ventricular outflow tract pressure gradient (62.91 mmHg) (Fig. 1C and Table 1). She had been suffering from shortness of breath during exercise for 16 years, which progressively worsened year by year. Additionally, she had a history of persistent atrial fibrillation for 4 years, and she had suffered from pulmonary embolism 2 years ago.

The proband's niece (HCM4 in Fig. 1A) was 37 years old and first diagnosed with HCM in a routine physical examination at the age of 22 years. She did not develop typical symptoms before the age of 35 years, and she had suffered from chest pain during exercise 2 years ago. She was diagnosed with non-obstructive HCM, with an interventricular septum thickness of 17.04 mm in our hospital (Fig. 1C and Table 1).

The proband's daughter (HCM6 in Fig. 1A) was 25 years old. She developed HCM at the age of 18 years. She showed a significant increase in interventricular septum thickness (16.82 mm) and left ventricular outflow tract pressure gradient (34.40 mmHg) (Fig. 1C and Table 1). She had been suffering from chest pain during exercise for 7 years.

The proband's mother and eldest sister had suffered from sudden cardiac death. The proband's father, second elder sister (HCM1 in Fig. 1A), nephew (HCM5 in Fig. 1A) and son (HCM7 in Fig. 1A) were 87, 65, 35, and 14 years old, respectively, and had normal echocardiographic measurements.

### 3.2 WES and bioinformatics analysis

We identified 377071 variants (326745 SNPs and 50326 INDELs) in HCM1, 1165887 variants (998525 SNPs and 167362 INDELs) in HCM2, 1159123 variants (992063 SNPs and 167060 INDELs) in HCM3, 1091480 variants (937739 SNPs and 153741 INDELs) in HCM4, 1288398 variants (1101867 SNPs and 186531 INDELs) in HCM5, 971888 variants (834069 SNPs and 137819 INDELs) in HCM6 and 1157935 variants (995657 SNPs and 162278 INDELs) in HCM7. The synonymous mutations in the human genome (NCBI37/hg19), the variants with a high mutation rate in the East-Asian population (MAF >0.001 in the Genome Aggregation Database) and the benign variants evaluated by InterVar program were filtered out. The retained variants (367 in HCM1, 419 in HCM2, 344 in HCM3, 383 in HCM4, 380 in HCM5, 374 in HCM6 and 409 in HCM7) were further analyzed according to Mendelian inheritance.

Since HCM often has an incomplete penetrance in adolescents,^[1]^ HCM1 (65-year-old) and HCM5 (35-year-old), but not HCM7 (14-year-old), were preliminarily selected as non-HCM controls. Following an inheritance pattern analysis, a total of 17 mutations in 17 genes were found heterozygous in patients with HCM (HCM2, HCM3, HCM4 and HCM6; Table 2). Among these mutations, a stop gained mutation, rs796925245 (NC_000001.11:g.201359630G>A) located in the troponin T2 (*TNNT2*) gene was predicted as a pathogenic mutation by InterVar program and found to be associated with the HCM phenotype in an autosomal dominant inheritance pattern. The same mutation was detected in 4 patients with HCM and HCM7, but was absent in HCM1 and HCM5. This stop gained mutation was predicted to result in a truncated troponin T protein (c.835C>T, p.Gln279Ter). Additionally, the effects of this nonsense mutation on protein coding were predicted to be deleterious by LRT, MutationTaster and fathmm-MKL programs, and were associated with familial isolated dilated cardiomyopathy in the Orphanet database (see Supplementary Digital Content, Table 1, Supplemental Content, which illustrates the effects of the mutations on protein coding predicted by 12 programs).

**Table 2:**
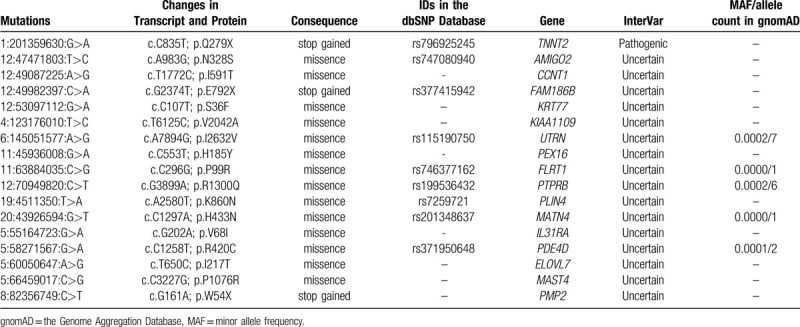
The Mutations Identified by Whole Exome Sequencing and Bioinformatics Analysis.

## 4 Discussion

The *TNNT2* gene is known to encode the cardiac muscle-specific isoform of troponin T, which binds the troponin complex to tropomyosin in the thin filament of sarcomere and plays an important role in the regulation of cardiac muscle contraction.^[22,23]^ The *TNNT2* gene is located on chromosome 1q32.1 and contains 17 exons. The mutations in the *TNNT2* gene account for 5% of patients with HCM who have been genotyped.^[3]^ WES is an efficient strategy to identify the pathogenic mutations that cause a rare mendelian disorder and reveal the genetic basis of the disease mechanisms.^[9,24]^ In the present study, using WES, a novel nonsense mutation in the *TNNT2* gene (rs796925245; g.1:201359630G>A, c.835C>T, p.Gln279Ter) was identified as the genetic pathogenic cause of HCM in a Chinese family with 4 HCM patients. The genetic form of the mutation is autosomal dominant. It is reported that left ventricular hypertrophy is rare in children with *TNNT2* gene mutations.^[25]^ HCM7, an adolescent with the Gln279Ter mutation in the *TNNT2* gene did not show the similar abnormal myocardial phenotype as other affected family members. However, it is worth noting that, in spite of having the normal ventricular wall values, a girl with the Arg278Cys mutation in the *TNNT2* gene was resuscitated after a cardiac arrest at the age of 17 years.^[26]^ Similarly, the Arg92Trp mutation in the *TNNT2* gene was found to be associated with a minimal left ventricular wall thickness, a low HCM penetrance and a high incidence of sudden cardiac death.^[27]^ Therefore, clinically unaffected *TNNT2* gene mutation carriers should be offered routine follow-ups and genetic counseling to prevent adverse cardiac events including sudden cardiac death.^[1]^ Additionally, the same pathogenic mutation in HCM2 (62-year-old female), HCM3 (50-year-old male), HCM4 (37-year-old female) and HCM6 (25-year-old female) resulted in different clinical manifestations, including age at diagnosis, symptoms, and degree and position of myocardial hypertrophy. Similarly, Garcia-Castro *et al.*^[28]^ found that a 60-year-old woman with the Arg278Cys mutation in the *TNNT2* gene had severe ventricular hypertrophy; however, her sister and daughter, who had the same mutation, had normal echocardiographic measurements. This may suggest that the mutations in the *TNNT2* gene are not closely associated with the severity of clinically demonstrable HCM.

The prevalence of HCM in China is 0.08%.^[5]^ In the present study, the low-frequency mutations (MAF <0.001 in the Genome Aggregation Database) in the East-Asian population, nonsynonymous mutations in human genome,^[29]^ non-benign variants in the InterVar program^[10]^ and deleterious mutations evaluated by 12 protein function predicting programs were considered as probable pathogenic mutations. Following screening and an inheritance pattern analysis, only the mutation Gln279Ter in the *TNNT2* gene was identified as a HCM-causing mutation in the pedigree.

The majority of the HCM-causing mutations in the *TNNT2* gene are missense.^[26,28]^ The mechanism underlying the functional effects of altered troponin T protein on cardiac muscle remains unclear. A previous *in vitro* study reported that the HCM-causing Ile91Asn mutation in rat troponin T resulted in faster thin filament movement.^[23]^ Furthermore, in a myotube expression system, 3 HCM-causing mutations (Ile79Asn, Arg92Gln and ΔGlu160) in the troponin T resulted in an increased energetic load on the heart.^[22]^ In the present study, a novel nonsense mutation in the *TNNT2* gene was identified as the HCM-causing mutation in a Chinese pedigree with HCM, which may result in a truncated troponin T (Gln279Ter). It was reported that truncated troponin T may lead to markedly reduced force production during cardiac contraction.^[30]^ It may be suggested that impaired troponin T creates stimuluses for HCM-causing compensatory ventricular hypertrophy by increasing myocardial motility and energetic load, and reducing force production during cardiac contraction.

In conclusion, a novel nonsense mutation (rs796925245; g.201359630G>A, c.835C>T, p.Gln279Ter) in the *TNNT2* gene was identified as the pathogenic mutation in a three-generation Chinese pedigree with HCM using WES. This study expanded the spectrum of HCM-causing *TNNT2* mutations and provided appropriate genetic counseling for a mutation carrier, who is still clinically unaffected.

## Acknowledgments

The authors would like to thank all patients and their family members for participating in this study, as well as the Genome Aggregation Database and the groups that have provided exome and genome variant data for this research. A full list of the contributing groups can be found at https://gnomad.broadinstitute.org/about.

Statement of Ethics

## Author contributions

**Data curation:** Guangyuan Gao, Weiwei Chen

**Funding acquisition:** Ping Yang

**Investigation:** Guangyuan Gao, Weiwei Chen

**Methodology:** Guangyuan Gao, Jinsha Liu, Xing Zhang and Max M. He

**Resources:** Guohui Liu, Yaliang Tong and Cuiying Mao

**Supervision:** Max M. He, Ping Yang

**Writing:** Guangyuan Gao

## Supplementary Material

Supplemental Digital Content
