# Supplementary material for: A novel nonsense mutation in TNNT2 in a Chinese pedigree with hypertrophic cardiomyopathy: A case report
Source: Medicine (Baltimore). 2020 Aug 21;99(34):e21843. doi: 10.1097/MD.0000000000021843 (PMC7447477; doi:10.1097/MD.0000000000021843)
Supplement: Supplemental Digital Content [file medi-99-e21843-s001.docx]

**Table S1.** The effects of the mutations on protein coding predicted by 12 programs

| Mutations | SIFT | PolyPhen-2_HDIV | Polyphen2_HVAR | LRT | MutationTaster | MutationAssessor | FATHMM | PROVEAN | MetaSVM | MetaLR | M-CAP | fathmm-MKL |
| --- | --- | --- | --- | --- | --- | --- | --- | --- | --- | --- | --- | --- |
| 1:201359630:G>A | - | - | - | D | D | - | - | - | - | - | - | D |
| 12:47471803:T>C | D | P | B | D | D | L | D | N | D | D | D | D |
| 12:49087225:A>G | T | B | B | N | N | N | T | N | T | T | T | D |
| 12:49982397:C>A | - | - | - | D | A | - | - | - | - | - | - | D |
| 12:53097112:G>A | D | D | D | - | D | M | D | D | D | D | D | N |
| 4:123176010:T>C | T | B | B | U | D | L | T | N | T | T | T | D |
| 6:145051577:A>G | T | B | B | D | D | L | T | N | T | T | T | D |
| 11:45936008:G>A | T | P | P | D | D | M | T | N | T | T | T | D |
| 11:63884035:C>G | D | D | D | D | D | M | T | D | T | T | D | D |
| 12:70949820:C>T | D | P | P | N | N | L | T | N | T | T | T | D |
| 19:4511350:T>A | T | B | B | N | P | N | T | N | T | T | - | N |
| 20:43926594:G>T | T | B | B | D | N | N | T | N | T | T | D | D |
| 5:55164723:G>A | T | B | B | U | D | M | D | N | T | D | T | N |
| 5:58271567:G>A | D | D | B | D | D | M | D | D | D | D | D | D |
| 5:60050647:A>G | D | P | B | N | D | L | T | D | T | T | T | D |
| 5:66459017:C>G | D | D | D | D | D | M | T | D | T | T | D | D |
| 8:82356749:C>T | D | D | D | D | D | H | T | D | T | T | - | D |
| Abbreviations: D = deleterious; T = tolerated; P = possible; B = benign; N = neutral; U = unknown; H = high; L = low; M = medium | | | | | | | | | | | | |
